# On the Use of Certain Caustics

**Published:** 1879-02-20

**Authors:** David W. Yandell

**Affiliations:** Professor of Surgery and Clinical Surgery, University of Louisville


### *ON THE USE OF CERTAIN CAUSTICS.*

[AN EXTRACT OF A CLINICAL LECTURE.]

BY DAVID W. YANDELL, M.D.,
Professor of Surgery and Clinical Surgery, University of Louisville.

*Gentlemen :* You have heard in a previous part of this course that caustics, so-called, are neither all of equal value nor of equal applicability in practice. In selecting a caustic, therefore, let your choice depend mainly on the work you propose to set it to do. What will fulfil all your expectations in one case may quite disappoint you in another; and yet the fault shall lie not with the caustic but with yourself—you having failed to select the right one, the one suited to that particular case. At many of our meetings you see me use one at least of half a dozen caustics which you will observe arranged on the surgical tray. Recently you witnessed me apply a hot iron to a bleeding surface. The wound was deep in the upper jaw; the blood oozed rather than ran from a number of small vessels; the simpler hemostatic means had failed, and of all agents the actual cautery was both the handiest and the most effective for this kind of hemorrhage in this particular locality. The same day you saw me destroy an old and ill-conditioned ulcer in the groin, following a bubo, with the Vienna paste. Every little while you have the opportunity to see me touch, and sometimes yourselves to touch, chancroids with the fuming nitric acid. This morning, after making a ghastly wound in order to remove an epithelioma from the side of a man's neck, I mopped the entire surface of the cut with a solution of chloride of zinc. Quite a number of times you have witnessed the application of pure carbolic acid to small surfaces of disease on different parts of the face. Now, the reason for varying the caustic in these different conditions is to be sought in the conditions themselves. Of the several agents I have mentioned, each, as I have remarked, is a valuable servant in its way. Neither possesses all the virtues nor all the drawbacks of the other. The hot iron was the easiest of application, as well as the most efficient for the arrest of oozing of blood consequent upon crushing through a diseased jawbone with a pair of forceps. Nitric acid is the handiest, the neatest, and altogether, I think, the best caustic for destroying chancroids. I don't say that the Vienna paste is better than any other agent for burning refractory ulcers in the groin, but I do say it is qdite as good. The solution of chloride of zinc is generally they ceded to be the most efficient application to surfaces, especially if conbe extensive, from which cancerous or suspicious tumors have been removed. It is thought to destroy with greater certainty than any other agent any parts of such growths as have escaped the knife. It has the advantages of being cheap, manageable and of easy application. It was a great favorite with my lamented friend, the late Prof. Bayless. I remember once seeing him apply it to the seat of an enormous encephaloid tumor, which he had removed from the abdominal walls of a woman. The wound healed quickly, and the disease did not return for four years after. It fell to me to do the second operation, but both the knife and the zinc this time proved unavailing—the wound did not heal, and the patient died. Carbolic acid has, in my hands, been all that I could desire for checking and causing to heal the small ulcerated surfaces which appear at times on the faces, particularly of persons who have light or sandy hair and very thin skins, and are haunted by the dread of cancer. To touch these little sores with the pure carbolic acid, which makes a white eschar, then to wait until this eschar falls off, and apply the acid a second or a third time, as the case may be, will usually cause the spots to heal. I have a patient, living now in Indianapolis, whose face is quite checkered with the white cicatrices denoting the seat of former sores. I have destroyed a number of small superficial nævi by the same agent.

To-morrow I shall attack, with yet a different caustic from any I have named, an epithelioma situated immediately over the malar bone. This agent, although not altogether new, has failed to attract, in our country at least, the attention which in my opinion its real merits fairly deserve. After a somewhat extensive trial, I feel warranted in saying that I am acquainted with nothing of this class which, in proper cases, is so efficient or trustworthy. It has quite a curious history, not unlike that of Ward's paste, which has enjoyed such reputation in the treatment of hemorrhoids. It, like the paste of the English charlatan, was purchased also from a quack. This time not by a vote of Parliament, but by Dr. Bell, an English physician visiting Paris, from Michel, a French charlatan who was plying his avocation there. The price paid was twenty-five thousand francs. Michel, it seems, used it for the destruction of external tumors generally, and declared that it was equal to the removal of almost any malignant tumor which was so situated that the paste could be applied. He daily attacked such malignant growths of the breast as were detached and solitary, and where the submaxillary glands were not involved, whether open or not, this making, as he asserted, no difference.

In dealing with tumors, the size say of a hen's egg, Michel laid the paste on the surface to the thickness of half an inch. This was followed by a rapid destruction of the tissues, which, the Frenchman says, is unaccompanied by pain after the first half hour or so. An oozing of clear watery fluid occurs, which is to be carefully sopped up. In twelve or fifteen hours the paste is to be removed, and a fresh application made. This is allowed to remain on for about the same length of time, when the work of the caustic is regarded as complete. The paste is wiped off, and the excavation treated as a simple sore. I have not followed these directions to the letter; but more of that presently.

When Dr. Bell became possessor of the secret, he found he had paid quite a round sum for a compound of strong sulphuric acid and finely powdered asbestos—three parts by weight of the former to one of the latter, thoroughly rubbed into a paste. I need not tell you that both these substances have been known to us all for a great while. It is perhaps, however, fair to Michel, the charlatan, to say that the merit of combining them for the purpose of making a caustic really belongs to him. Ward's celebrated paste, you remember, consisted of the simple confection of pepper, an officinal preparation of the earliest dispensatory. Dr. Bell, with that old-fashioned honesty and liberality which is so characteristic of our English progenitors, made public the formula of the French quack's paste, which, excepting the inert matter of the asbestos, is identical with the carbo-sulphuric paste so much used by Ricord in the treatment of venereal sores. Dr. Bell's revelation soon found its way to this country, and, seeing it, I determined to put the paste on trial. No druggist in this city had the powdered asbestos, but Dr. Davis, one of the teachers in the School of Pharmacy here, suggested that the French soapstone was chemically identical with the asbestos, whereupon a paste was made of it and the acid. From that time until now I have used this combination. It is applied in the same way with the Vienna paste, the thickness of the layer depending upon the amount of tissue it is desired to destroy, varying, say from one-eighth to four-eighths of an inch in depth. It is spread upon the diseased surface with a small wooden spatula, or with the blade of a knife. It dries at the end of a few hours, the time varying with the depth of the layer and the amount of blood which oozes during its application. When used on a large surface, it gives rise to considerable and prolonged suffering, so much indeed that I seldom deny to the patient some anæsthetic. The paste should always be freshly-made. I rarely keep it an hour. Like the Paris plaster it is best to apply it on a dry day, lest, as you will be told in another room, the great affinity of the acid for the water, causing it to absorb the moisture from the atmosphere, should lessen the power of the paste. For fear it should run upon the surrounding healthy parts, it is well to encircle the portion which you intend to destroy either with a good layer of tallow or collodion or adhesive plaster. The patient must endure the pain which succeeds upon his recovery from anæsthesia as best he may, unless it be so severe as, in your opinion, to call for an opiate. I have had patients tell me that they suffered for half a day. The paste when dry remains, unless colored by blood, a brownish white. It adheres for a varying length of time; the longer it does so, the more thorough does it prove its application to be, and the better for the patient, since the work of repair goes on undisturbed beneath it. When it falls off, or grows loose and is removed, the sore is found, in the more fortunate cases, to be healthy, when simple dressing only will be required till cicatrization is complete. Should the ulcer, on the other hand, not present a satisfactory appearance, the paste must be re-applied.

I could tell you of many quite striking cures of both lupus devorans and epithelioma which the Frenchman's caustic has accomplished in my hands; but two must suffice for the present.

A gentleman from a distant part of this state, with an epithelioma involving almost the whole of his lower lip, came to me, now four years ago. I destroyed the diseased parts most thoroughly by the paste. To do this involved the loss of the entire lip. Several applications were required. The patient recovered in good time, and has remained well. He covers his disfigurement with a large moustache, and prevents drooling by wearing a piece of lint where his lip once was. If the choice of means of cure had been left in this case to me, I should have selected the knife; but the patient would not hear to this, and I was driven to the paste. I am sure he suffered more than he would have done if he had allowed me to use the quicker means, but the final result would have been no better, perhaps not so good.

A lady, who had a lupus covering the whole of one cheek, had tried three different caustic applications for its relief. The sulphuric-acid paste thickly spread over the diseased surface effected a complete cure in a month. She has remained well for three years.

When you return to your homes and meet with cases of lupus and epithelioma, and your minds turn to caustics for their relief, please remember the estimate I place upon Michel's paste in these affections—the only ones in which I have used it.—*Louis. Med. News.*

